# Supplementary material for: Evaluation of Immune Response to Mucosal Immunization with an Oral Probiotic-Based Vaccine in Mice: Potential for Prime-Boost Immunization against SARS-CoV-2
Source: Int J Mol Sci. 2023 Dec 22;25(1):215. doi: 10.3390/ijms25010215 (PMC10779021; doi:10.3390/ijms25010215)
Supplement: Supplementary file 1 [file ijms-25-00215-s001.zip › ijms-2721761-supplementary.pdf]

Supplementary Material

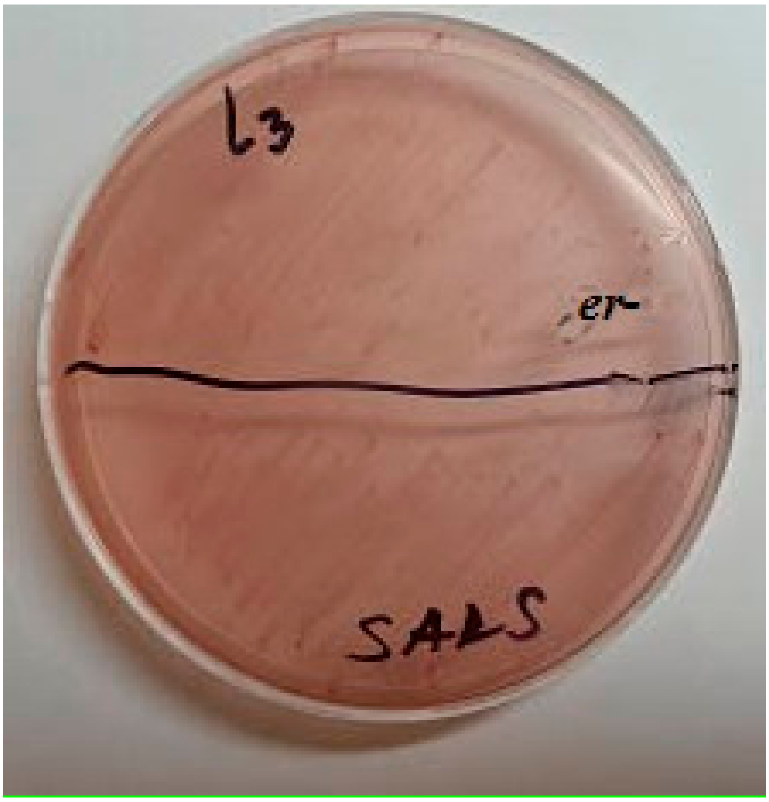

Figure S1. *E.faecium* L3 (L3) and L3-SARS-CoV-2 (SARS) were plated on azide agar without erythromycin(er-) and incubated for 24 hours at 37° C. Both cultures grow on the agar.

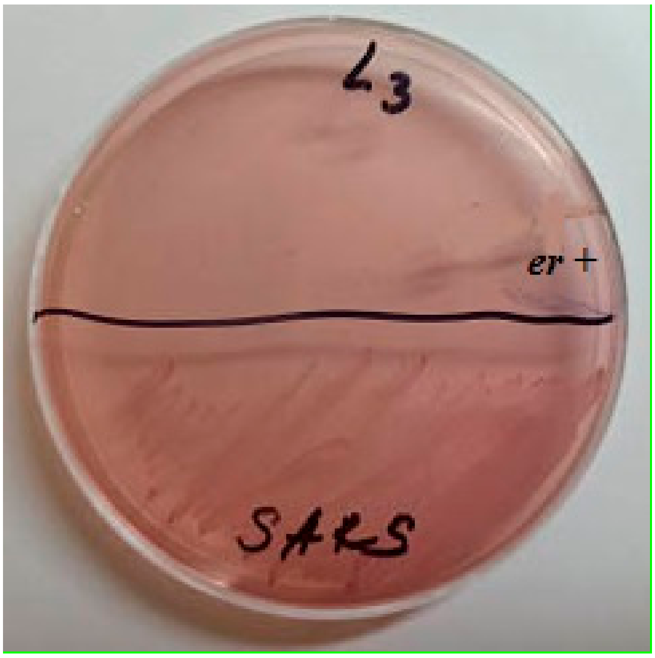

Figure S2. *E.faecium* L3 (L3) and L3-SARS-CoV-2 (SARS) were plated on azide agar with 5 µg / ml erythromycin (er+) and incubated for 24 hours at 37° C. Only L3-SARS grows on the agar.

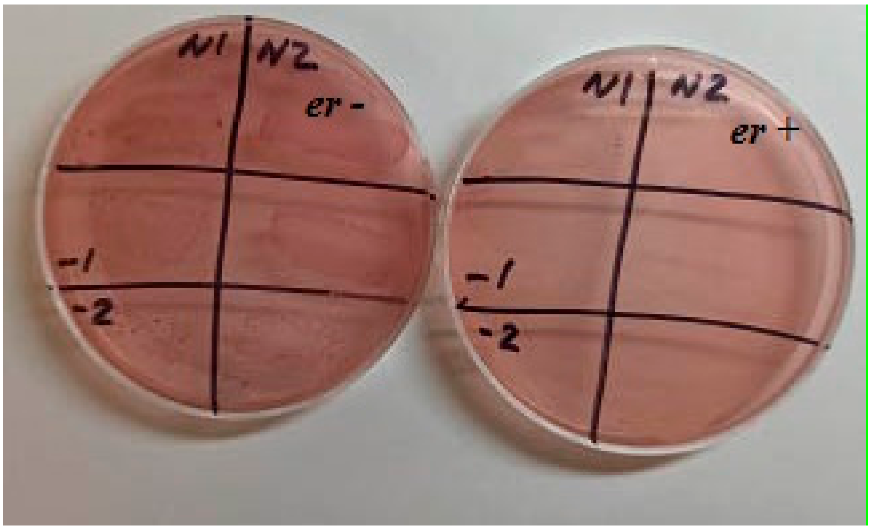

Figure S3. A suspension of fecal samples from intact mice (n=2) was plated on antibiotic-free azide agar (er-) and agar supplemented with 5 µg / ml erythromycin (er+). Enterococci from the natural intestinal microbiota of mice grow only on antibiotic-free azide agar.

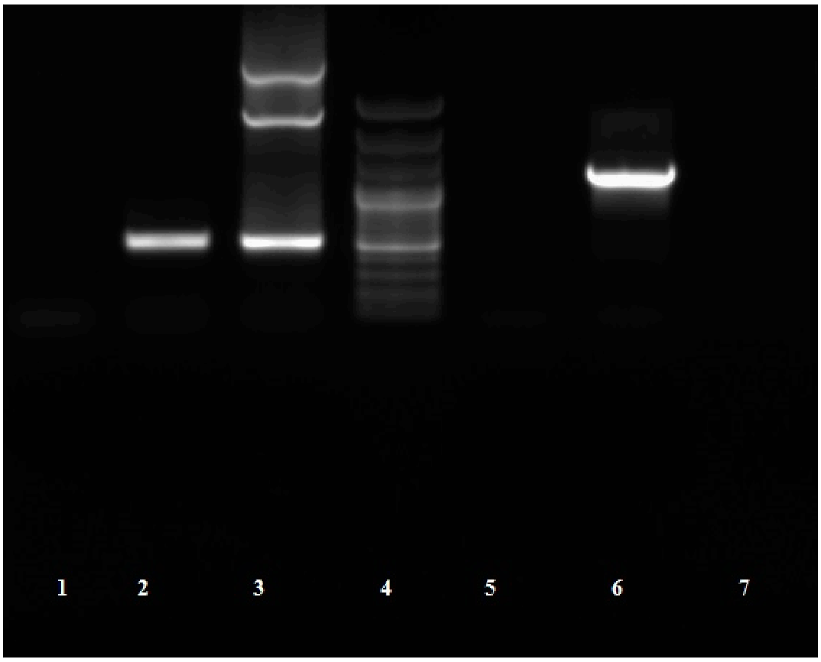

Figure S4. An example of PCR analysis of the DNA isolated from a typical colony grown on the azide agar supplemented with 5 µg / ml erythromycin after plating a suspension of feces samples from vaccinated mice.

DNA amplification:

1- PCR product using template DNA from L3 agar colony and K1 и K2 as primer sequences;

2-PCR product using template DNA from L3-SARS agar colony and K1 и K2 as primer sequences;

3- PCR product using erythromycin plasmid containing viral insert as template and K1 и K2 as primer sequences;

4- 100 bp ladder DNA marker (100–3000 bp);

5- PCR product using template DNA from L3 agar colony and B1 и K2 as primer sequences;

6-PCR product using template DNA from L3-SARS agar colony and B1 и K2 as primer sequences;

7- PCR product without added DNA.

K1: TTGCATATGGGTTTCCAACCCACT forward

K2: GTAGAATTCGTTGTTGACATGTTCA reverse

B1: TGAGTGAACCACAGCCAGAA forward

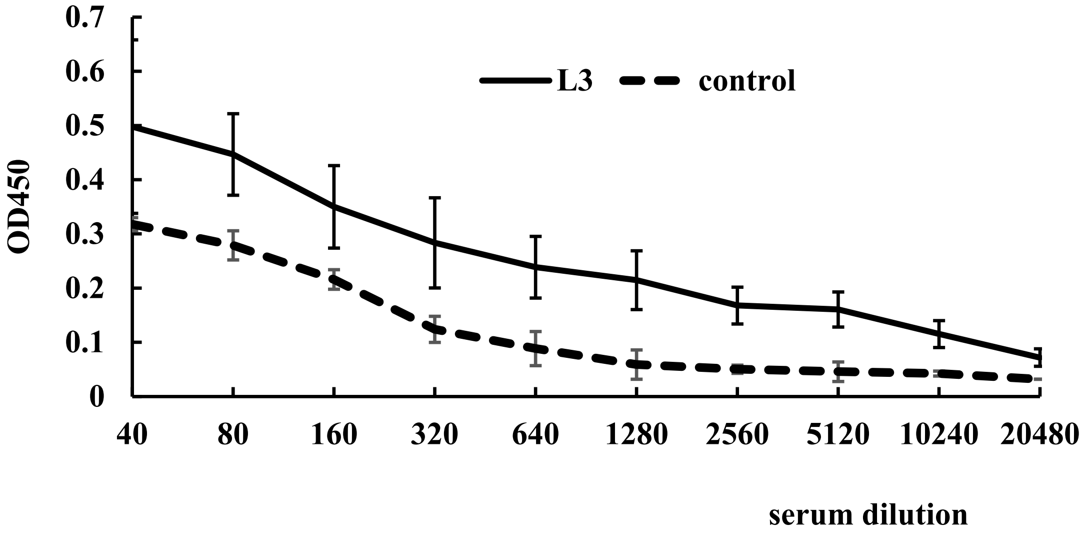

Figure S5. Sera titration curves from two control groups against protein S in ELISA. A comparative analysis included sera from untreated mice (control) and mice orally given probiotic *E. faecium* L3 (L3). The graph depicts mean optical density at 450 nm (OD450) ± SEM, (n=6).
